# Polyclonal antibody against the DPV UL46M protein can be a diagnostic candidate

**DOI:** 10.1186/1743-422X-7-83

**Published:** 2010-04-29

**Authors:** Liting Lu, Anchun Cheng, Mingshu Wang, Jinfeng Jiang, Dekang Zhu, Renyong Jia, Qihui Luo, Fei Liu, Zhengli Chen, Xiaoyue Chen, Jinlong Yang

**Affiliations:** 1 Avian Diseases Research Center, College of Veterinary Medicine of Sichuan Agricultural University, Ya, an, Sichuan, China; 2 Key Laboratory of Animal Diseases and Human Health of Sichuan Province, Ya, an, Sichuan, China; 3 Epizootic Diseases Institute of Sichuan Agricultural University, Ya, an, Sichuan, 625014, China; 4 Chongqing Academy of Animal Science, Chongqing, 402460, Chongqing China

## Abstract

**Background:**

The duck plague virus (DPV) UL46 protein (VP11/12) is a 739-amino acid tegument protein encoded by the *UL46 *gene. We analyzed the amino acid sequence of UL46 using bioinformatics tools and defined the main antigenic domains to be between nucleotides 700-2,220 in the *UL46 *sequence. This region was designated UL46M. The DPV *UL46 *and *UL46M *genes were both expressed in *Escherichia coli *Rosetta (DE3) induced by isopropy1-β-D-thiogalactopyranoside (IPTG) following polymerase chain reaction (PCR) amplification and subcloning into the prokaryotic expression vector pET32a(+). The recombinant proteins were purified using a Ni-NTA spin column and used to generate the polyclonal antibody against UL46 and UL46M in New Zealand white rabbits. The titer was then tested using enzyme-linked immunosorbent assay (ELISA) and agar diffusion reaction, and the specificity was tested by western blot analysis. Subsequently, we established Dot-ELISA using the polyclonal antibody and applied it to DPV detection.

**Results:**

In our study, the DPV UL46M fusion protein, with a relative molecular mass of 79 kDa, was expressed in *E. coli *Rosetta (DE3). Expression of the full *UL46 *gene failed, which was consistent with the results from the bioinformatic analysis. The expressed product was directly purified using Ni-NTA spin column to prepare the polyclonal antibody against UL46M. The titer of the anti-UL46M antisera was over 1:819,200 as determined by ELISA and 1:8 by agar diffusion reaction. Dot-ELISA was used to detect DPV using a 1:60 dilution of anti-UL46M IgG and a 1:5,000 dilution of horseradish peroxidase (HRP)-labeled goat anti-rabbit IgG.

**Conclusions:**

The anti-UL46M polyclonal antibody reported here specifically identifies DPV, and therefore, it is a promising diagnostic tool for DPV detection in animals. UL46M and the anti-UL46M antibody can be used for further clinical examination and research of DPV.

## Background

Duck plague virus (DPV) is a pantropic, generalized infection virus, which can induce an acute, septic, contagious, and lethal disease in ducks, geese, swans, and all members of the family Anatidae of the order anseriformes. The mortality rate of infected adult ducks is up to 90%; therefore, DPV is considered one of the most severe blights in the waterfowl breeding industry worldwide [1].

The DPV genome is composed of linear, double-stranded DNA with 64.3% guanine-plus-cytosine content, which is higher than any other reported avian herpesvirus in the subfamily Alphaherpesvirinae [2]. Although DPV was previously grouped in the subfamily Alphaherpesvirinae, it was classified as an unassigned virus in the Herpesviridae family according to the Eighth International Committee of Taxonomy of Viruses [3-5]. However, the molecular characteristics of DPV remain largely unknown. Following the development of molecular biology, the research has focused on the molecular biology of the etiological agent of DPV, especially its genome atlas and encoding proteins, rather than the generation and distribution of the virus in its host, the construction and morphogenesis of DPV, and the prevention and diagnosis of DPV [6-11]. To date, studies on the genomic organization and nucleotide sequence of DPV lag behind other members of the Herpesviridae family and no reports have been published concerning the DPV gene *UL46*. DPV gene transcription can be classified into 3 types: immediate-early (IE), early (E), and late (L) [12]. *UL46*, which is not essential for virus replication, is a late transcription gene of the herpesviruses. As the phosphorylated product of *UL46 *translation, the UL46 protein (VP11/12) plays an important role in enhancing the efficiency of αTIF (VP16)-mediated α gene expression and initiates α gene transcription through an unknown mechanism of action. Generation of an antibody against DPV UL46 will further research on the function and bionomics of DPV.

Considering that *UL46 *may be expressed at a low level or fail to be expressed in a prokaryotic system due to its long sequence (2,220 bp), we selected peptide fragments with high antigenicity by predicting the hydrophilicity and antigenicity of UL46, designated UL46M, in addition to using the complete *UL46 *gene. *UL46 *and *UL46M *were expressed in *E. coli *Rosetta (DE3) by constructing the prokaryotic recombinant expression plasmids pET32a(+)/UL46 and pET32a(+)/UL46M. The DPV UL46M fusion protein had a relative molecular mass of 79 kDa, while expression of the full *UL46 *gene failed. The recombinant protein was used to generate the polyclonal antibody against UL46M in rabbits. ELISA and western blot identified anti-UL46M antibody with a high titer and strong specificity, and the antibody was preliminarily applied in the specific detection of DPV by Dot-ELISA. The results provide a compact foundation for research on the function of UL46 and its use in the diagnosis of DPV.

## Results

### Analysis of hydrophilic and antigenic indices of the DPV UL46 protein

**Figure 1:**
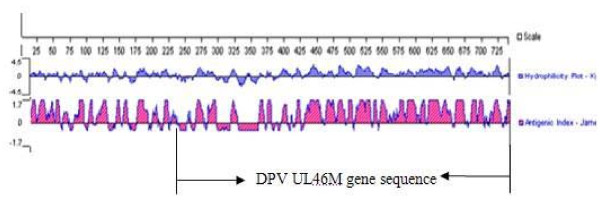
**Analysis of hydrophilicity and antigenic index of DPV UL46 protein**. The hydrophilicity and antigenic index of DPV UL46 protein were analyzed by DNAstar6.0. Then the main antigen regions UL46M was selected on the basis of the analysis result and was expressed with the complete *UL46 *gene.

Generally, the expression of the main antigenic regions of the protein was prioritized in order of increasing immunogenicity and specificity of the corresponding antibody. Therefore, we analyzed the hydrophilic and antigenic indices of UL46 and selected 507 amino acids (site, 233-739) (Figure 1) as the main antigenic region for expression to avoid lack of expression, as was the case for the full *UL46 *gene.

### Gene amplification, cloning, and sequencing

**Figure 2:**
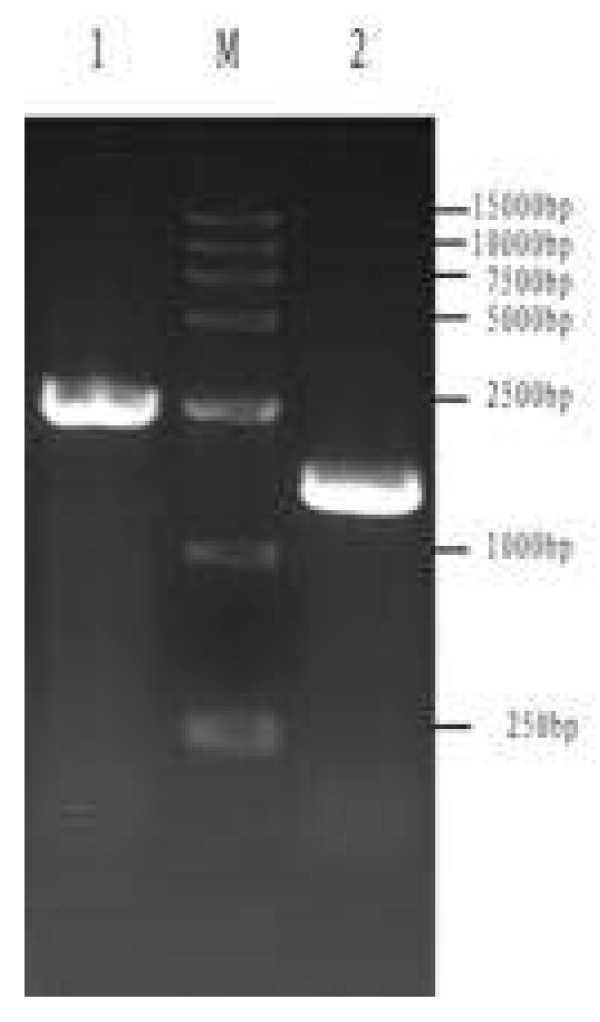
**PCR products of the fragments of DPV *UL46M *and *UL46M *gene**. Lane 1, PCR product of DPV *UL46*; Lane M, DNA marker; Lane 2, PCR product of DPV *UL46M*.

**Figure 3:**
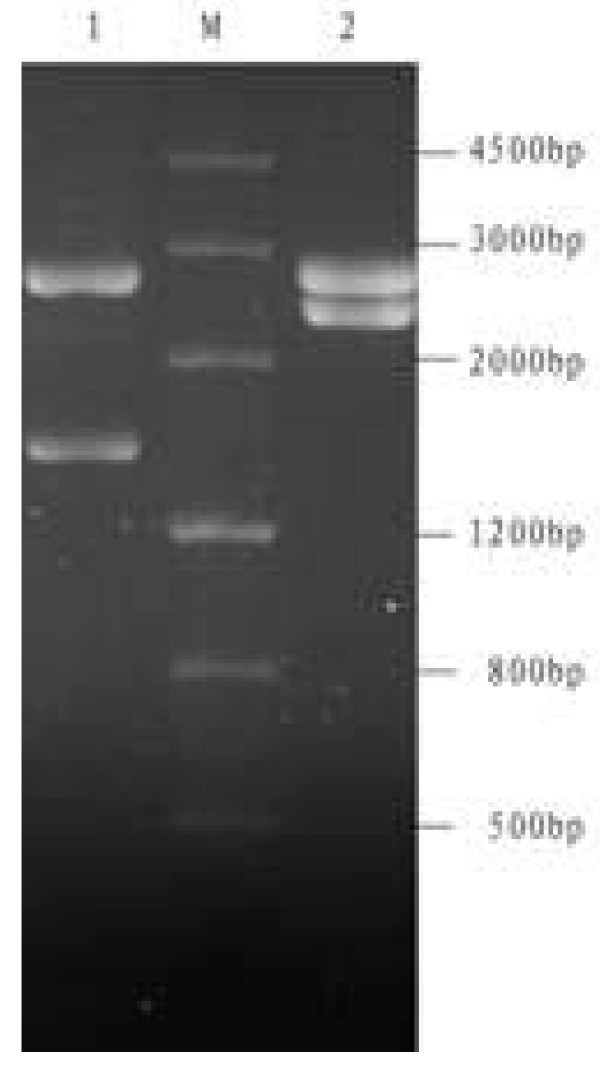
**DPV *UL46 *and *UL46M *gene encoding DNA sequences were cloned into pMD18-T cloning vector**. The recombinant plasmids were digested with two restriction enzymes. Lane 1, *Bam*HI and *Xho*I generating two restriction fragments of *UL46M*; Lane M, DNA marker; Lane 2, *Bam*HI and *Xho*I generating two restriction fragments of *UL46*.

Two regions of DPV, approximately 2,500 bp and 1,500 bp in *UL46 *and *UL46M*, respectively, were amplified by PCR (Figure 2, lane 1 and lane 2). The PCR products were digested with *Bam*HI and *Xho*I restriction enzymes and the open reading frames (ORFs) were inserted into the pMD18-T vector to construct the cloning vectors pMD18-T/UL46 and pMD18-T/UL46M. The recombinant plasmids were then confirmed by DNA sequencing and restriction digests (Figure 3, lane 2 and lane 1). The sequencing results showed that there were no nucleotide errors in the amplified *UL46 *and *UL46M *gene fragments (data not shown).

### Expression and purification of recombinant protein

**Figure 4:**
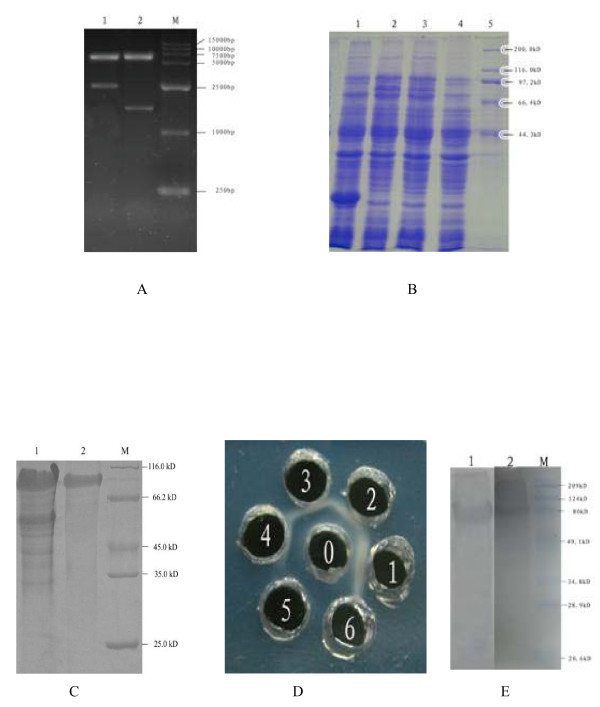
**A. DPV *UL46 *and *UL46M *gene encoding DNA sequences were cloned into pET32a (+) procaryotic expression vector**. The recombinant plasmids were digested with two restriction enzymes. Lane 1, *Bam*HI and *Xho*I generating two restriction fragments of *UL46*; Lane M, DNA marker; Lane 2, *Bam*HI and *Xho*I generating two restriction fragments of *UL46M*. B. Induction of the 6× His-tagged UL46M fusion protein in *E. coli *plasmid pET32-a (+)/UL46M was transformed into bacteria. Bacteria were grown in the absence (lane 4) or the presence (lane2 and lane 3) of IPTG. Induced pET32-a (+) was as control (lane 1). Molecular mass marker (in kDa) were shown to the right (lane 5). C. The recombinant UL46M fusion protein was purified by Ni-NTA affinity chromatography. Lane 1, unpurified recombinant UL46M fusion protein; Lane 2, purified recombinant UL46M fusion protein; Lane M, protein marker. D. Detection result of the antisera titer by agar diffusion reaction. The result of the agar diffusion reaction of the anti-UL46M antiserum showed the largest dilution multiple of the positivation was 1:8. 1-6.1, 2, 4, 8, 16, 32-fold diluted antisera; 0. DPV. E. Analysis of the antibody specificity by western blot The result revealed that the purified recombinant protein was recognized by the anti-UL46M rabbit IgG and showed a specific signal at the expected size (79 kDa). No positive signal was observed when using the negative control sera (date not shown). Lane 1, western blot of anti-DPV IgG with the UL46M protein; Lane 2, western blot of anti-DPV UL46M IgG with the UL46M protein; Lane M, prestained protein marker.

The *UL46 *and *UL46M *gene fragments were subcloned from pMD18-T/UL46 and pMD18-T/UL46M into the prokaryotic expression vector pET32a(+) using *Bam*HI and *Xho*I and were confirmed by restriction enzyme analysis (Figure 4a, lane 1 and lane 2). The newly formed vectors were designated pET32a(+)/UL46 and pET32a(+)/UL46M, respectively. To express *UL46 *and *UL46M*, the pET32a(+)/UL46 and pET32a(+)/UL46M plasmids were transformed into competent *E. coli *Rosetta (DE3) cells. However, only a distinct band approximately 79 kDa, corresponding to the expected UL46M protein size, was obtained after a 4-h induction with 0.7 mM isopropy1-β-D-thiogalactopyranoside (IPTG) (Figure 4b, lane 2 and lane 3). Expression of the complete *UL46 *gene was not successful. Expressed protein was not detected in the induction of *E. coli *Rosetta (DE3) cells carrying an empty pET32a(+) vector (Figure 4b, lane 1) or in the negative control without induction (Figure 4b, lane 4). The recombinant UL46M fusion protein was purified by Ni-NTA affinity chromatography (Figure 4c, lane 2) based on the 6× His tag present at its N-terminal. The density of the UL46M fusion protein was 3.09 mg/mL by the Bradford method.

### Verification of the character of the polyclonal antibody

① Detection of the antiserum titer by agar diffusion reaction. The highest titer of the agar diffusion reaction of the anti-UL46M antiserum from the 6 rabbits showed that the largest positive dilution multiple was 1:8 (Figure 4d). The highest titer of anti-UL46M antibodies from the 6 rabbits as determined by ELISA was 1:819,200 (Table 1). The pre-immune serum was used as a negative control. ② Analysis of antibody specificity by western blot. The result revealed that the anti-UL46M rabbit IgG antibody recognized the purified recombinant protein, showing a specific signal at the expected size (79 kDa) (Figure 4e, lane 2). No positive signal was observed when using the negative control sera (date not shown), indicating that the recombinant protein induced an immunological response and that the antisera had a high level of specificity. This suggests that the antiserum is suitable for DPV detection in clinical diagnoses. Additionally, these results were supported by the results of the western blot with anti-DPV IgG and the UL46M protein (Figure 4e, lane 1).

**Table 1:** The results of ELISA (OD_450 nm/630 nm_)

*Dilution proportion*	*① Antisera*	*② Normal sera*	*①/②*
1:400	1.721	0.428	4.021
...	...	...	...
1:409600	0.688	0.102	6.745
1:819200	0.441	0.087	5.069
1:1638400	0.302(<0.4)	0.053	

The values were the mean results of three parallel experiments, when ①/② ≥2.1 *and *① ≥ 0.4, the ELISA result was positive. The pre-innune sera was the negative control.

### Detection of DPV by Dot-ELISA

**Figure 5:**
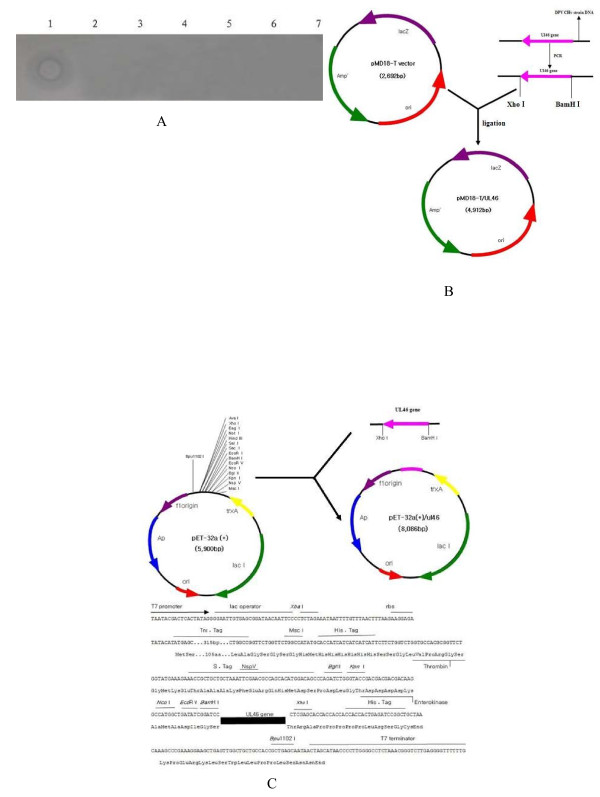
**A. Detection of DPV by Dot-ELISA**. The preliminary application of the polyclonal antibody against DPV UL46M was the establishment of Dot-ELISA to detect DPV. The result showed stronger positive signal with the liver sample of DPV, while negative with DHV-1, *E. coli *(O1), SE, RA, P. multocida and normal saline. 1. DPV, 2. DHV-1, 3. *E. coli *(O1), 4. SE, 5. RA, 6. P. multocida, 7. normal saline (negative). B. Schematic diagram of the UL46 ORF cloned into the pMD18-T cloning vector. The fragment of UL46 digested with *Bam*HI and *Xho*I was cloned into cloning vector pMD18-T at 16°C overnight using DNA Ligation Mix to generate recombinant cloning plasmid named pMD18-T/UL46. C. Construction of the recombinant expression plasmid pET32a (+)/UL46. The fragment of UL46 digested with *Bam*HI and *Xho*I from pMD18-T/UL46 was cloned into a 6× His-tagged prokaryotic expression vector pET32a(+) at *Bam*HI and *Xho *sites and designated it as expression vector pET32a(+)/UL46.

The preliminary application of the polyclonal antibody against DPV UL46M was in the detection of DPV by Dot-ELISA. Thus, the samples were prepared on a nitrocellulose (NC) membrane and the anti-UL46M IgG and HRP-labeled goat anti-rabbit IgG antibodies were used for DPV detection. The square matrix test determined that the suitable dilution of anti-UL46M IgG was 1:60 and that of HRP-labeled goat anti-rabbit IgG were 1:5,000. Dot-ELISA showed a stronger positive signal for DPV in the liver sample and was negative with duck hepatitis virus-1 (DHV-1), *E. coli *(O1), *Salmonella enteritidis *(SE), *Riemerella anatipestifer *(RA), *Pasteurella multocida*, and normal saline, as shown in Figure 5a.

## Discussion

The *UL46 *gene is not evolutionarily conserved among the different Herpesvirus subfamilies. *UL46 *is only conserved in alphaherpesviruses such as Herpes simplex virus type 1 (HSV-1) and is not present in beta- and gammaherpesviruses such as human cytomegalovirus (HCMV) and Epstein-Barr virus (EBV), respectively (13-15). Although *UL46 *is not essential for virus replication, the formation of plaque bacteriophage can be effected in the absence of *UL46 *[16,17]. VP11/12, the phosphorylated product of the translated *UL46 *gene, plays an important role in enhancing the efficiency of αTIF (VP16)-mediated α gene expression and in initiating α gene transcription [18]. Therefore, the research conducted here on DPV UL46 and corresponding antibody characteristics revealed significant theoretical and practical value for understanding the molecular mechanism of DPV.

For the preparation of the anti-UL46 rabbit antibody, 2 factors had to be considered. First, the collecting of main antigens was the key for a gene, especially for the longer fragment, and the stronger hydrophilic and antigenic regions were important for maintaining the immunoreactivity of the antigen. Thus, we selected the more hydrophilic and antigenic regions of the DPV *UL46 *gene, namely, the 507 amino acid N-terminal (233-739 site), as the main UL46 antigen in addition to the complete *UL46 *gene to ensure greater specificity and a higher titer of the corresponding antibody. Second, it was difficult to extract UL46 from infected cells and the relative molecular weight of UL46 was only 81.8 kDa. Additionally, the weaker antigenicity of UL46 may not elicit a stronger immune response in rabbits that were already immunized. Therefore, the prokaryotic expression vector pET32a(+) was chosen to express the UL46M fusion proteins in *E. coli*.

In our study, the *E. coli *host cells BL21 (DE3), BL21 (DE3) plysS, and Rosetta were all used to express *UL46 *and *UL46M*. The results identified that the expression level of *UL46M *was similar among the 3 host cells, and the full *UL46 *gene failed to express. Temperature was a major influence on the expression level, compared with inducing time and IPTG density. In addition, the recombinant protein was expressed within inclusion bodies. Since the inclusion body did not possess biological activity, the protein was redissolved and renatured prior to inoculation. His-tagged UL46M was expressed using pET32a(+), which was convenient for recombinant protein purification. In addition, the His tag did not influence the structure or function of UL46M due to its small molecular weight, and even inclusion bodies were beneficial in increasing the stability of product by preventing proteolytic degradation of the protein.

The Dot-ELISA has become a new addition to the diagnostic arsenal against microbes, contagious and parasitic diseases, because it is easy to use, is economical, requires small antigen dosage, and the results are easy to interpret. Therefore, we established the Dot-ELISA for DPV detection using the anti-DPV UL46M polyclonal antibody. The result revealed polyclonal antibody specificity for DPV; thus, we concluded that this anti-UL46M antibody could be used to diagnose DPV.

We employed a double wavelength (450 nm/630 nm) to detect the optical density of samples in order to decrease light interference caused by scratches or fingerprints on the 96-well microtiter plate. Considering that antigen purity was not 100% and that the polyclonal antibody was based on many epitopes, the purified IgG was used to avoid nonspecific binding during titer quantification, antibody specificity determination, and application of Dot-ELISA. The results revealed that the anti-UL46M rabbit antibody prepared in our study was of high titer and specificity.

## Conclusions

In conclusion, the preparation of the specific anti-UL46M rabbit antibody established a foundation for further research on the biological activity and molecular mechanism of *UL46*. This can be extended to the qualitative and quantitative analysis of the UL46 protein using immunofluorescent and immunochemical techniques, thus providing useful tools for studying the structure and function.

## Methods

### Analysis of hydrophilic and antigenic indices of DPV UL46protein

The NCBI BLASTN and ORF Finder servers were used to find an ORF. Then, the hydrophilic and antigenic indices of DPV UL46 were analyzed using the DNAstar6.0 software (DNASTAR Inc., USA), by using the predicted amino acid sequence of the complete ORF to obtain the main antigenic domain of UL46 (UL46M).

### Preparation of DPV DNA

DPV was propagated in Duck Embryo Fibroblasts (DEFs) that were cultured in Minimum Essential Medium (MEM) (Invitrogen, Carlsbad, CA) containing 10% fetal bovine serum (FNS) (Invitrogen, Carlsbad, CA) and 1-2% penicillin and streptomycin at 37°C. After virus infection, MEM supplements with 2-3% FBS and 1-2% penicillin and streptomycin were used. The virus particles were harvested when the cytopathic effect reached 75%. Cell lysates containing DPV were subjected to 3 freeze-thaw cycles and were then stored at -70°C until use. The extraction of DPV DNA was performed as described previously [19].

### PCR amplification of the *UL46 *and *UL46M *genes

We identified and isolated the major antigenic domains of UL46, which were designated as UL46M, in conjunction with the full-length *UL46 *gene. Based on the constructed DPV CHv-strain genomic library, the primer sequences for PCR amplification of the DPV *UL46 *and *UL46M *genes were synthesized by TaKaRa (Dalian, China) as follows: (A) the full *UL46 *gene: forward primer (P1) 5'-**GGATCC**ACGGTGATGTCGTCCAGG-3' and reverse primer (P2) 5'-**CTCGAG**GCGTCTTTGGTTTGTCGTAA-3', and (B) the *UL46M *gene: forward primer (P1) 5'-**GGATCC**CCGCTGGATCTTATGGTT-3' and reverse primer (P2) 5'-**CTCGAG**TTATTTCCCAAATGACAGTCT-3' [20]. The *Bam*HI and *Xho*I sites that were used to clone the PCR fragment are bolded in the primer sequences. The primers were dissolved in ultrapure water to a concentration of 20 pmol/μL. The PCR amplifications contained 12.5 μL of 2× Taq PCR MasterMix (TianGen, Beijing, China), 1 μL (20 pmol/μL) of each primer, 1 μL of template (10 ng/μL), and ultrapure water to a total reaction volume of 25 μL. The PCR cycle parameters were as follows: (A) the complete *UL46 *gene: 5 min at 95°C and 30 cycles of 1 min at 94°C, 1 min at 59°C, 2 min 40 s at 72°C, and a final extension time of 10 min at 70°C; (B) the *UL46M *gene: 5 min at 95°C and 30 cycles of 1 min at 94°C, 1 min at 56°C, 1 min 50 s at 72°C, and a final extension time of 10 min at 70°C. The amplified products were visualized by gel electrophoresis (10 g/L agarose gel containing 5 μL/100 mL goldview).

### Construction and identification of the cloning plasmids pMD18-T/UL46 and pMD18-T/UL46M

The purified PCR products were digested with restriction enzymes *Bam*HI and *Xho*I, purified, and ligated into the correspondingly digested cloning vector pMD18-T at 16°C overnight using DNA Ligation Mix. The subsequently generated recombinant cloning plasmids were named pMD18-T/UL46 and pMD18-T/UL46M, respectively (e.g., *UL46*, Figure 5b). The recombinant plasmids were transformed into *E. coli *DH5α cells, and the transformants were cultured at 37°C on Luria-Bertani (LB) solid medium (1.0% sodium chloride, 1.0% tryptone, 0.5% yeast extract, and 1.5% agars) for 16 h. The masculine clones were collected and grown in liquid LB medium (1.0% sodium chloride, 1.0% tryptone, 0.5% yeast extract, and 100 μg/mL ampicillin) at 37°C for 12 h. The recombinant plasmids were verified by PCR and designation under the above condition. Each clone was then selected and sent to TaKaRa for sequencing. Then we performed the nucleotide homology comparison with the public sequence (GenBank: EU195108) available in NCBI GeneBank using DNAMAN and blast tools.

### Construction and identification of the recombinants pET32a(+)/UL46 and pET32a(+)/UL46M

After confirmation of the sequencing results, pMD18-T/UL46 and pMD18-T/UL46M plasmids were digested with *Bam*HI and *Xho*I and purified using a TIANprep Mini Plasmid Kit (TianGen). We then cloned the respective fragments into the 6× His-tagged prokaryotic expression vector pET32a(+) at the *Bam*HI and *Xho*I sites and designated them as expression vector pET32a(+)/UL46 and pET32a(+)/UL46M (e.g., *UL46*, Figure 5c) [21]. The selected positive colonies were identified by PCR and designated under the above condition.

### Prokaryotic expression of the DPV *UL46 *and *UL46M *genes

*E. coli *Rosetta (DE3) cells transformed with the prokaryotic expression plasmids pET32a/UL46 and pET32a/UL46M were cultured in presence of ampicillin (100 μg/mL) in LB medium with vigorous shaking at 37°C until an A_600 _of 0.4-0.6. The expression of the recombinant fusion proteins was induced by addition of 0.7 mM/L IPTG and further shaken at 37°C for 4 h. After induction, the cells were harvested by centrifugation at 6,000 rpm for 10 min at 4°C and lysed in 5× sodium dodecyl sulfate-polyacrylamide gel electrophoresis (SDS-PAGE) loading buffer (0.313 M Tris-HCl (pH 6.8), 50% glycerol, 10% SDS, and 0.05% bromophenol blue with 100 mM DTT). The uninduced control and the vector control cultures were analyzed in parallel.

### Purification of the recombinant proteins by Ni-NTA

As described above, 4 g of wet weight cells from a 1-L culture was harvested by centrifugation at 6,000 rpm for 10 min, and the pellet was suspended in 20 mL lysis buffer (20 mM Tris-HCl buffer (pH 8.0) containing 100 mM NaCl, 1.0 mM phenylmethyl sulfonylfluoride (PMSF), and 1.0 mg/mL lysozyme). The suspension was incubated for 30 min at 4°C with stirring and was then pulse-sonicated on ice (30 s working and 30 s resting on ice; Vibracell VCX 600 sonicator; 600 watt max, Sonics & Materials Inc., USA) until the sample was clear. Sonication was performed to lyse cells and release intracellular protein. The resulting cell lysate was centrifuged at 12,000 rpm for 30 min (AM50.14, Thermo electron Co.). The collected pellet was dissolved in deionized water and analyzed by 12% SDS-PAGE. The recombinant protein was purified using the Ni-NTA Spin Column kit, according to the manufacturer's instructions. The density of the recombinant protein was detected using the Bradford method and stored at -80°C until use [22].

### Production of the rabbit polyclonal antibodies

Six male New Zealand white rabbits were immunized using purified recombinant DPV UL46M protein according to Hu et al. [23]. One milliliter of pre-immune sera was collected from the ear margin of each rabbit as the negative control. Each rabbit was injected with 0.5 mg antigen mixed with complete Freund's adjuvant in a 1:1 ratio on the back and proximal limbs. After 1 week, the rabbits were subsequently injected 3 times with the antigen (1.0 mg/rabbit) mixed with incomplete Freund's adjuvant at intervals of 1 week. Two weeks after the fourth injection, the rabbits were sacrificed and the antisera was harvested from the arteriae carotis and stored at -80°C until use.

### Purification of the antisera

The rabbit IgG fraction was precipitated from the harvested rabbit polyclonal antisera in saturated ammonium sulfate according to Walker et al. [24]. Then, the IgG fraction was purified by High-Q anion-exchange chromatography following the manufacturer's instructions using a DEAE-Sepharose column (Bio-Rad) and was analyzed on a 12% SDS-PAGE gel.

### Identification of the polyclonal antibody

① Detection of the antisera titer by agar diffusion reaction. One gram of agar was dissolved in buffered saline prepared by the addition of 0.85 g of sodium chloride to 100 mL of distilled water. The mixture was heated, cooled down to 55°C, and poured into the plates to a thickness of 2 mm. The agar was then perforated with 3 mm-diameter holes that held approximately 100 μL of solution. Thirty microliters of 1-, 2-, 4-, 8-, 16-, and 32-fold diluted antisera was added into the peripheral apertures and DPV was added into the central aperture. The plate was diffused at 37°C for 24 h. The largest dilution multiple of the sediment band identified the antibody titer. ② Detection of the titer of anti-UL46M rabbit antibody by ELISA. A 96-well microtiter plate (Nunc, Denmark) was coated with 100 μL (0.01 mg/L) purified DPV in sodium bicarbonate buffer (pH 9.6) and incubated at 37°C for 1 h and then at 4°C overnight. The plate was blocked with 100 μL of blocking solution (1% BSA in PBS) for 1 h at 37°C and washed 3 times with PBST (0.05% Tween 20 in PBS). Subsequently, 100 μL of a 2 multiple (1:400 to 1:819,200) dilution of purified anti-UL46M IgG was added and incubated at 37°C for 1 h. The plate was washed and incubated for 1 h at 37°C with 100 μL of a 1:5,000 dilution of anti-HRP-labeled goat anti-rabbit IgG diluted, washed again and detected with 100 μL of 3,3',5,5'-tetramethylbenzidine (TMB)-H_2_O_2 _for 30 min at room temperature. The reaction was stopped by the addition of 50 μL of 30% H_2_SO_4_. 20 min later, and the optical density (OD) was determined at 450 nm/630 nm double wavelength using a Bio-Rad model 860 plate reader (Bio-Rad, CA, USA). The normal rabbit sera and PBST were used in parallel as the negative control and blank, respectively. When the OD value of the anti-sera was ≥0.4 and the ratio with normal sera was ≥2.1, the result was positive. The largest positive dilution multiple was the antisera titer. ③ Analysis of antibody specificity by western blot. To characterize the specificity of the antibody, western bolt analysis was performed according to standard procedure [19]. Then, the DPV UL46M protein was separated on a 12% SDS-PAGE gel. Following electrophoresis, the gel was immersed in transfer buffer (0.24% Tris-HCl, 1.153% glycine, and 15% methanol, pH 8.8) and electro-blotted onto polyvinylidene difluoride (PVDF) membrane at 100 V for 1.5 h. The membrane was incubated in blocking buffer (5% BSA in the PBS buffer) for 1 h at 37°C. The membrane was incubated with purified anti-UL46M IgG (1:200 dilution) overnight at 4°C after three washes with PBST buffer (0.2% Tween 20 in PBS, pH 7.4). The membranes were incubated with HRP-labeled goat anti-rabbit IgG (Bodter) in a 1:5,000 dilution for 1 h at 37°C. The membrane was developed with DAB substrate buffer following PBST washes and terminated by washing in distilled water. Western blot of anti-DPV IgG for UL46M was performed accordingly.

### Detection of DPV by Dot-ELISA

① Animal test. One day old ducks were infected with one of DPV, duck Hepatitis Virus type 1 (DHV-1), *E. coli *(O1), SE, RA, and *P. multocida*, and the livers from the dead ducks were obtained as the antigen species, while normal saline was used as the negative control in parallel. ② Sample preparation. Aseptic PBS was added in a 1/3 (w/v) ratio to the samples, which were then grinded into homogenate and centrifuged at 8,000 rpm for 10 min at 4°C after freeze-thawing 3 times at -20°C. The supernatant was collected as the antigen species for detection by Dot-ELISA, and detection using negative and blank controls was conducted in parallel. ③ Detection of Dot-ELISA. The NC membrane was cut to optimal size and the sample spot was marked using a pencil. The membrane was then saturated in ddH_2_O for 10 min and dried at room temperature. Five microliters of treated samples, at dilutions greater than 1:100, were loaded onto the NC membrane at the previously marked locations (spotting a small amount of sample and drying at room temperature each time), followed by drying the NC membrane completely at room temperature. The NC membrane was blocked for 1 h at 37°C using blocking solution (1% BSA in PBS) and washed 3 times (5 min each) with PBST (0.05% Tween 20 in PBS). Subsequently, the membrane was incubated with a 1:60 dilution of rabbit anti-UL46M IgG with 0.1% BSA in PBS overnight at 4°C, and washed the following day. The membrane was further incubated for 1 h at 37°C with anti-HRP-labeled goat anti-rabbit IgG diluted 1:5,000 in PBS and developed using a DAB substrate buffer at room temperature until an amethyst signal was observed. Thorough washing in ddH_2_O terminated the reaction. The negative and blank controls were conducted in parallel. Distinct spots with consistent structures indicated a positive result, while fuzzy spots with structural anomalies or the lack of a spot indicated a negative result.

## Competing interests

The authors declare that they have no competing interests.

## Authors' contributions

LL carried out most of the experiments and wrote the manuscript. AC and MW critically revised the manuscript and the experiment design. JJ, DZ, RJ, QL, FL, ZC, XC and JY helped with the experiment. All of the authors read and approved the final version of the manuscript.
